# Application of Genetic Studies to Flow Cytometry Data and Its Impact on Therapeutic Intervention for Autoimmune Disease

**DOI:** 10.3389/fimmu.2021.714461

**Published:** 2021-08-31

**Authors:** Valeria Orrù, Maristella Steri, Francesco Cucca, Edoardo Fiorillo

**Affiliations:** ^1^Institute for Genetic and Biomedical Research, National Research Council (CNR), Sardinia, Italy; ^2^Department of Biomedical Sciences, University of Sassari, Sassari, Italy

**Keywords:** drug development, immune profiling, autoimmune diseases, GWAS, flow cytometry

## Abstract

In recent years, systematic genome-wide association studies of quantitative immune cell traits, represented by circulating levels of cell subtypes established by flow cytometry, have revealed numerous association signals, a large fraction of which overlap perfectly with genetic signals associated with autoimmune diseases. By identifying further overlaps with association signals influencing gene expression and cell surface protein levels, it has also been possible, in several cases, to identify causal genes and infer candidate proteins affecting immune cell traits linked to autoimmune disease risk. Overall, these results provide a more detailed picture of how genetic variation affects the human immune system and autoimmune disease risk. They also highlight druggable proteins in the pathogenesis of autoimmune diseases; predict the efficacy and side effects of existing therapies; provide new indications for use for some of them; and optimize the research and development of new, more effective and safer treatments for autoimmune diseases. Here we review the genetic-driven approach that couples systematic multi-parametric flow cytometry with high-resolution genetics and transcriptomics to identify endophenotypes of autoimmune diseases for the development of new therapies.

## Introduction

The human immune system is a magnificent biological network of specialized cells and their soluble products that can recognize and tolerate “self” and harmless symbionts while mounting responses to “non-self”, including the panoply of harmful pathogens. Immune cell subtypes are the pivotal determinant to maintain immunity and minimize the loss of tolerance that can result in autoimmunity. Because immune cells must orchestrate and mount responses to a variety of insults, their circulating levels are extensively regulated by exposure to environmental factors, and in particular by pathogen infection. Nevertheless, in the last 10 years the assessment of genetic effects on circulating levels of immune cells and their surface proteins (collectively referred as immune cell traits) has revealed that they are on average ~40% heritable (1, 2), meaning that a high percentage of variability in their levels is regulated by genetic differences among individuals. The high heritability of immune cell traits has prompted us and others (1–6) to assess the genetic contribution to their variability through systematic genome wide association studies (GWAS) in general populations. Overall, hundreds of associated variants have been identified.

More recently, a GWAS-based approach on cytometric data has also been applied, albeit in a small sample size, to assess the genetic control of changes in immune cell levels after exposures such as influenza vaccination (7). This type of analysis is likely to become increasingly common and performed in much larger sample sets, for example to assess cellular response to the Sars-Cov-2 vaccine. There are three key requirements to use the powerful and unbiased tool of GWAS to understand how the immune cells are genetically regulated and to identify overlaps with autoimmune disease risk. The first is a very detailed measurement of a broad spectrum of cell types, encompassing innate and adaptive immunity, by assessing their activated, regulatory, inflammatory and maturation states. The second is high-resolution characterization of genetic variability in the same individuals. The third requirement is generating or obtaining summary statistic data of autoimmune disease GWAS to establish overlap with immune cell GWAS. The sample size of immune cell GWAS is pivotal to infer a full range of genetic associations. Indeed, while a few thousand individuals, like those assessed in the immune cell trait GWAS performed thus far, identify genetic associations of common variants with relatively large effect size, tens of thousands of individuals must be analyzed to discover genetic associations with rare variants, and those with smaller effect size (8). Further broadening the spectrum of associated variants through substantial increases in the sample size evaluated in immune cell trait GWAS will thus be important to identify many more overlapping associations with disease. Of particular interest are multiple overlaps with the same immune trait and disease, strengthening the evidence for a causal relationship and thereby increasing the power to identify therapeutic targets.

**Figure 1:**
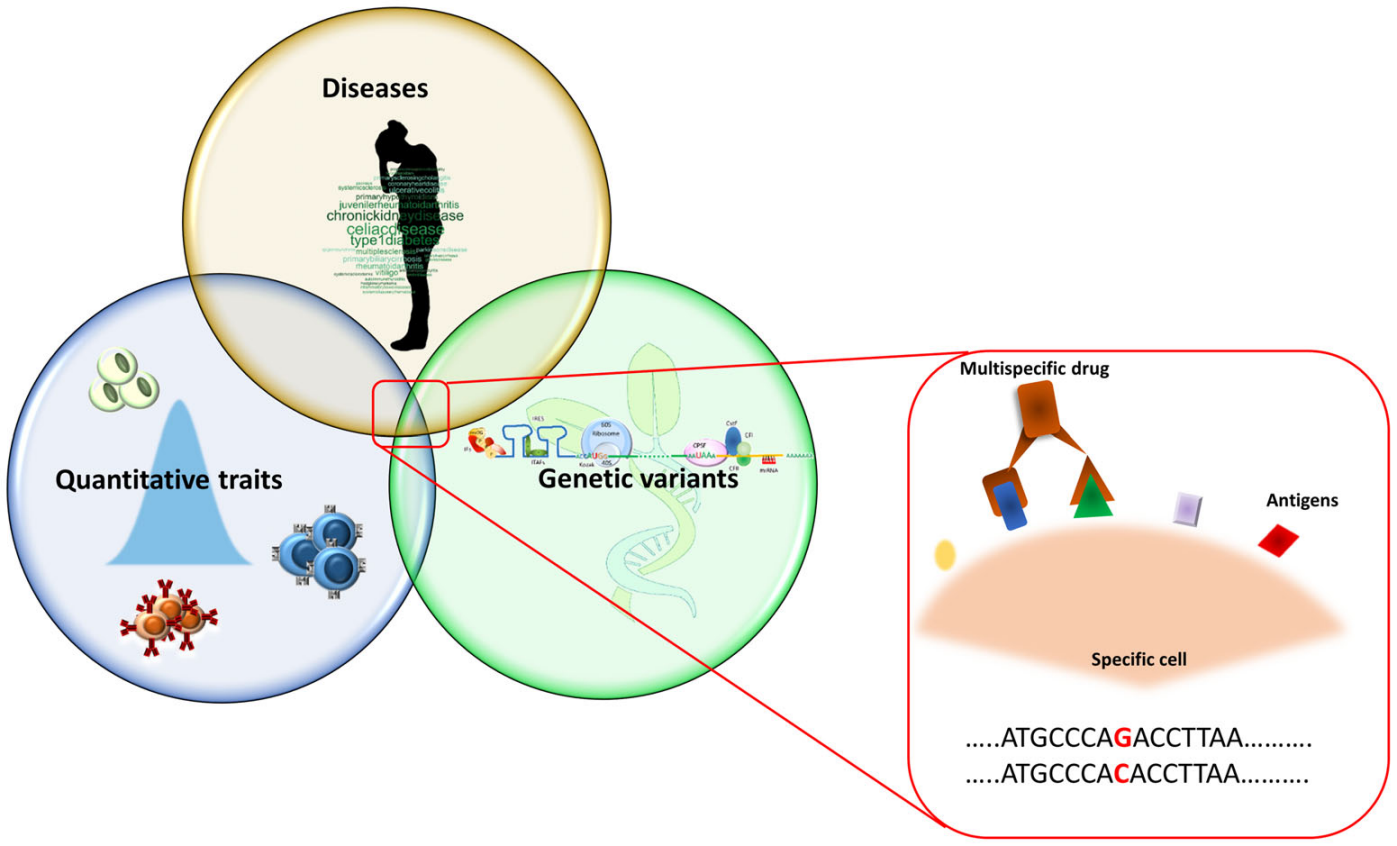
Overview of the study approach. The picture summarizes the research of overlapping genetic associations that initially consists in the identification of genetic signals regulating immune cells in the general population. These signals are then compared with those associated with diseases with the aim to identify the genetic variants both affecting immune cells and disease risk. The identified immune cells can be considered as potential drug target on which to act pharmacologically. Being more frequently detectable by more than one antigen expressed simultaneously on their surface, these cells can be targeted by multi-specific drugs (binding two or more specific antigens).

Focusing on immune cell traits, the most common technique to systematically measure cell subpopulations as well as surface or intracellular proteins, is flow cytometry. Routinely used for functional studies, flow cytometry is now becoming the starting point to identify DNA variants associated with immune traits and, in turn, those variants that are also associated with risk of disease (hereafter referred to as “overlapping genetic associations”). This approach can identify cell types, molecules and pathways implicated in disease pathogenesis and provide prime candidates for more specific and efficacious therapeutic intervention (**Figure 1**). The potential of the genetic-driven approach in the research and development of new drugs is supported by the observation that 73% of studies supported by genetic evidence targeting the disease pathway were successful in Phase II clinical trials compared with 43% of studies without such genetic link (9). Nevertheless, genetic studies provide only a powerful substrate for experimental elucidation of disease mechanisms. Thus, causality must be confirmed by functional experiments *in vitro* and *in vivo*, which, in the context discussed here, are essential to clarify the biological mechanisms underlying the overlapping associations with specific immune cell traits and disease risk and formulate robust therapeutic hypotheses that are critical to the success of new drug research and development programs.

In particular, genetic associations of quantitative cellular traits and autoimmune diseases are more likely to give rise to biological investigations that are truly related to the causal biology of diseases than epidemiological surveys of environmental factors and observational studies of phenotypic variables that can often highlight second-order phenomena that are a consequence, and not a cause, of the disease. In this sense, although epidemiological evidence clearly indicates that environmental factors should play a very important role in the regulation of the immune system and contribute to the risk of autoimmune diseases, their precise identification is complicated by numerous factors and remains largely elusive. In contrast, genetics represents a more direct, powerful, and unbiased tool to generate robust hypotheses about disease-causing mechanisms that need to be further investigated with functional studies to identify and validate therapeutic targets (10).

We turn to an outline of the evolution of flow cytometry; the proper generation of flow cytometry data; and the application of GWAS to flow cytometry-based immune profiling to identify new drug targets.

## Flow Cytometry

**Figure 2:**
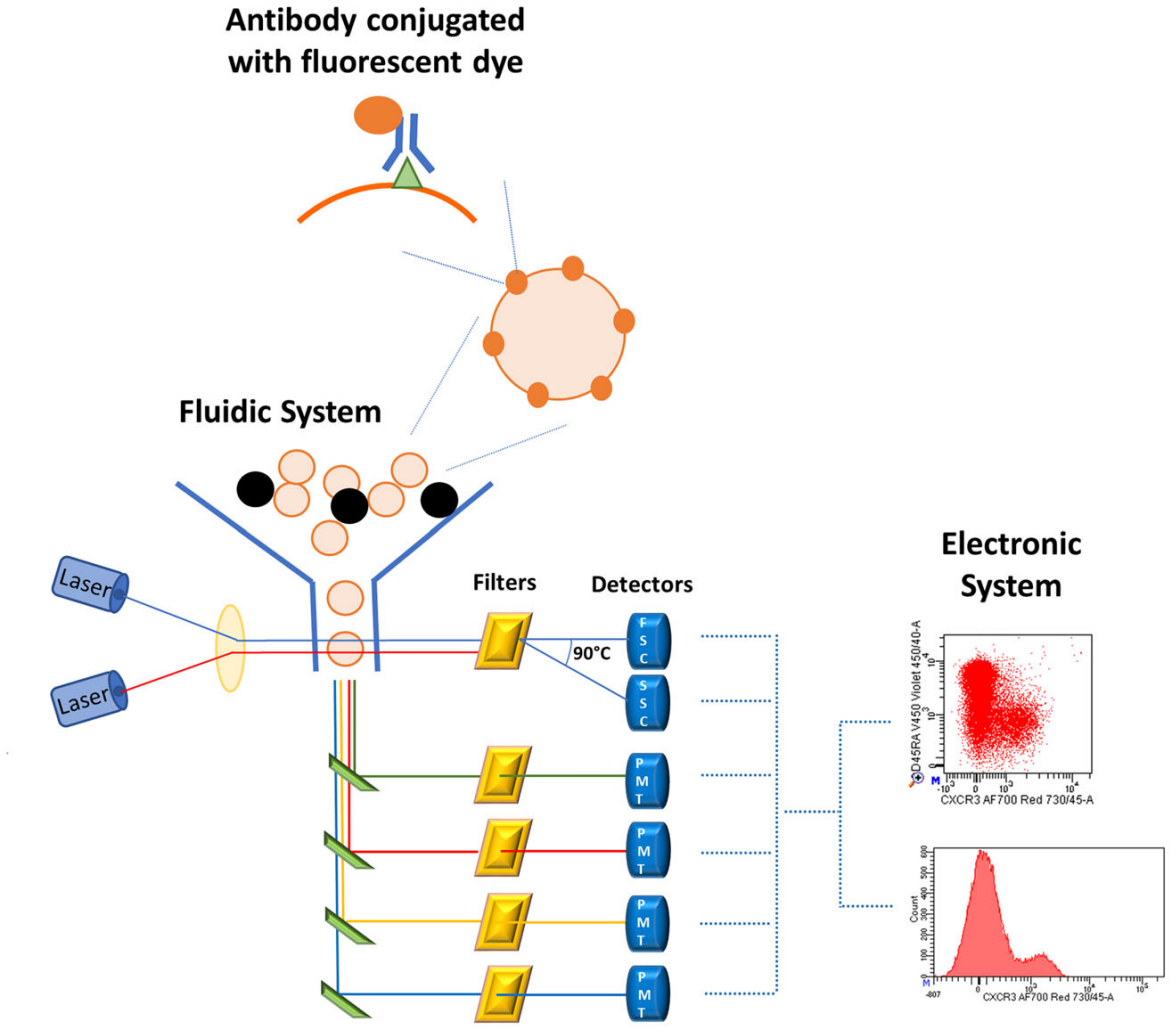
Schematic representation of flow cytometry system. Cells stained with fluorescent-conjugated antibodies are aligned in the fluidic system where they encounter one or more laser beams which excite the fluorescent dyes bound to the cells. The fluorescent antibodies emit at a specific wavelength and the emission is proportional to the amount of antigen-antibody complex. The emission arrives to the optical system, consisting of filters, mirrors, and photomultiplier tubes (PMTs), which enhance and improve the signal. Finally, the electronic system converts the fluorescent emission in electronic signals visualized by histograms or bi-dimensional plots.

The role of flow cytometry (**Figure 2**) in scientific research and clinical practice is increasing dramatically and only a marginal part of its potential is currently being used. However, while this technique is very useful if applied correctly and with appropriate checks, it can lead to incorrect conclusions if not. We will dedicate the next two sub-sections to describe this technology and some tips for using it properly.

### Flow Cytometry From Its Inception to Today

Flow cytometry development (11) was accompanied by important evolution of its applications in several scientific fields, including not only immunology but also hematology, cancer, microbiology, and physics. For instance, flow cytometric oncology panels are widely used to diagnose hematologic malignancy, especially B cell lymphoproliferative disorders, based on disproportion of kappa and lambda immunoglobulin light chains that are expressed on membrane surface of B cells. Indeed, a kappa-lambda ratio higher than 3:1 or lower than 1:3 is respectively considered evidence of monoclonality and diagnostic for B cell lymphoproliferative disorders (12).

In microbiology, flow cytometry allows the detection of microbes, their viability and distribution within cells that can have profound impact in infection diagnosis (13). Furthermore, in some countries, application of flow cytometry to microbiology has been routinely applied to water quality analysis (14).

Improving performance and processivity and increasing the number of parameters measured simultaneously by flow cytometers is the major challenge for flow cytometry companies. For instance, to reduce the time of sample processing and the variability of data acquisition, an acoustic focusing chamber characterized by high frequency sound produced by a piezoelectric device were applied to a flow cytometer (15). This system generates a standing wave in the sample capillary, which can align cells in the center of the flux even when the original cell concentration is high.

To increase the number of antibodies assessed simultaneously, an alternative cytometry-based technique, namely “CyTOF” (cytometry by time-of-flight), was developed about ten years ago. Similarly, to flow cytometry, antigens are recognized by antibodies labeled with heavy metal isotopes (instead of fluorochromes) which, as in mass spectrometry, are detected based of on their time-of-flight (16). CyTOF is more expensive than classical flow-cytometry, require longer period of time to process each sample, making this method unsuitable for processing large amounts of samples in a short time, but it can detect more than 100 parameters per cell simultaneously.

**Figure 3:**
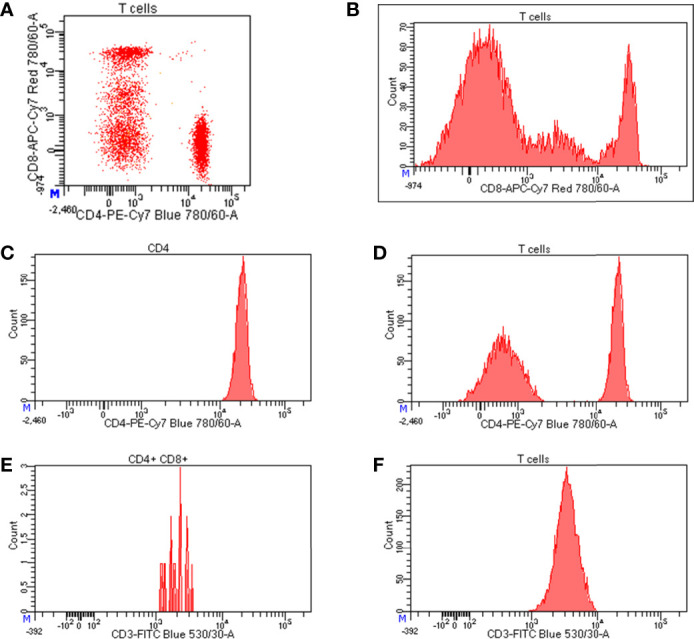
Representation of flow cytometry data. **(A)** Bi-dimensional visualization of data (dot plot) where each axis represents an antigen; **(B)** histograms representing the expression level of CD8 on T cells; starting from left to right, the first peak corresponds to CD8 negative T cells, the second peak represents cells expressing intermediate level of CD8, whereas the third peaks indicates highly positive cells for CD8 expression; **(C)** normal distribution of CD4 expression on CD4 positive cells; **(D)** bimodal distribution of CD4 expression on T cells where the peak on the left corresponds to CD4 negative T cells, while the peak on the right represents CD4 positive T cells; expression levels of CD3 on **(E)** a poorly represented cell population (CD4+CD8+ T cells) and **(F)** a well-represented cell population (T cells).

Flow cytometry has also become the starting point for big data projects such as genetic studies of thousands of immune cell traits, and single cell transcriptomic and proteomic measurements. Moreover, the simultaneous assessment of several fluorochrome-conjugated antibodies (17) (destined to increase soon) in thousands of individuals allows the identification of very rare cell subsets and of new cell types never previously described, but at the same time, it increases the difficulty of analysis of the enormous amount of data generated. Indeed, to visualize an n-dimensional flow data, 12×n×(n−1) bi-dimensional plots would be needed, so that, for instance, an experiment assessing 20 antibodies would require 12×20×(20−1)=190 bi-dimensional plots to display all marker combinations. Thus, data produced by the latest generation flow cytometry and CyTOF need to be visualized in alternative ways, departing from the classical bi-dimensional plots and histograms (**Figures 3A, B**).

**Figure 4:**
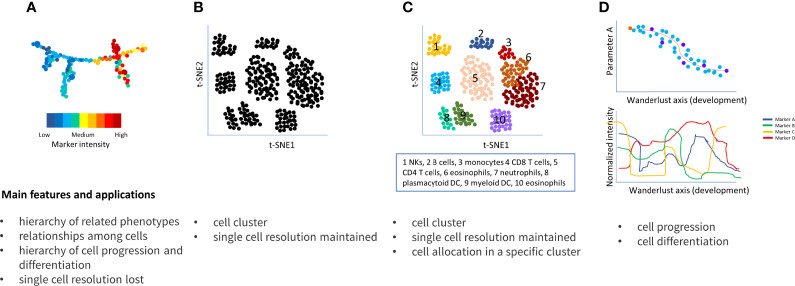
Main approaches to resolve flow cytometry data complexity. **(A)** SPADE connects clusters of multidimensional data in a progressive dendrogram. Cluster sizes correlate with the number of cells within the cluster. The heat map indicates the intensity of each cluster based on the median intensities of a protein marker in each cell node; **(B)** t-SNE detects cluster corresponding to cell population, similar cell are placed close together reflecting their proximity in high-dimensional space; **(C)** vi-SNE, ACCENSE, DensVM and Phenograph are evolution of t-SNE and similarly visualized; in particular, Phenograph is able to assign each cell into a specific cluster; **(D)** Wanderlust orders cells into a trajectory corresponding to their developmental stages.

Two of the most popular algorithms to reduce the complexity of this big amount of data and to identify populations of interest are SPADE (spanning-tree progression analysis of density-normalized events) (18) and t-SNE (t-stochastic neighbor embedding) (19). Both resolve high-dimensional data into a single bi-dimensional plot, the former visualizing cell clusters through dendrograms and the latter by scatter plots, so that the closer the cell clusters are, the more similar they are (**Figures 4A, B**).

SPADE and t-SNE do not allocate every cell to a specific cluster, nevertheless, automated clustering algorithms such as ACCENSE (20), DensVM (21), viSNE (22), to mention only a few of them, can help to solve this issue. However, these algorithms do not consider the entire dimension of the dataset; to address this, PhenoGraph was developed (**Figure 4C**) (23).

Another algorithms, named Wanderlust (24) is particularly useful to study temporal developmental cell relationships by generating a trajectory, for example ranging from hematopoietic stem cell through the mature status of the assessed cells (**Figure 4D**). Both PhenoGraph and Wanderlust represent each cell by a node that is linked to its neighbors by edges; thus, phenotypically similar cell clusters are visualized by interconnected nodes, namely “neighborhoods” or “communities” of cells (25).

In case of comparisons among two or more groups (such as patients and controls), Citrus is another useful tool to identify differential cell clusters and response features among the assessed groups that could be predictive of different experimental or clinical endpoints of interest (26). For instance, comparing unstimulated *vs* stimulated peripheral blood mononuclear cells, Citrus was able to identify 117 cluster features (out of 465) which differed between the two conditions.

### Guides to Correct Flow Cytometry Analysis

Before starting data collection and analysis, a strict process of quality checks and controls is pivotal to obtain reproducible and robust results. The most important steps can be summarized as follows.

1) *Panel set-up*. Increasingly, a number of common antigens are found to be expressed in cells whose biological role is supposed to be radically different. For instance, Schuh and colleagues described the uncommon co-expression of CD3 (receptor complex characterizing T cells) and CD20 (characterizing B cells) in a small subset of circulating lymphocytes that are especially frequent in the cerebrospinal fluid of multiple sclerosis patients (27). This underlines the need for several cell antigens simultaneously assessed as mandatory for a comprehensive immune cell analysis and for the discovery of rare cell populations that may nevertheless be potentially relevant in disease predisposition. However, the simultaneous assessment of many antigens requires a complex panel set-up that implies careful selection of antigen-fluorochrome combinations. A general role for fluorochrome-antigen selection is to use weak fluorochromes for highly expressed antigens and, vice-versa, bright fluorochromes for weakly expressed antigens. This allows detection of weak signals while keeping on scale brighter ones and minimizing the spillover of one fluorochrome into those having close emission wavelength. The mathematical correction of this spillover is called compensation and is an extremely important step that must be done before analyzing data to avoid misleading interpretations (28).
2) *Processing of samples*. The protocol to be followed and the time between sample collection and processing are pivotal to ensure reproducibility of flow data, especially for specific cells and antigens. For instance, monocytes are prone to modify their morphology and the expression of some antigens on their surface, including the costimulatory molecules CD80 and CD86 (29), while platelets are subject to very fast modifications and activation. Thus, this blood component should be processed within minutes after blood collection (30, 31). Similarly, the stability of antibodies is important: the Lyotube™ technology, employing lyophilized predefined cocktails of antibodies, is more stable than corresponding liquid formats, thus minimizing fluorochrome decay and allowing reduction of potential operator-dependent variations (1, 32).
3) *Sample freezing*. Freezing is known to damage some antigens and cell types, such as myeloid derived suppressor cells (defined as CD66b+ and CD15+, HLA-DRdim and CD14−) that are not detectable in previously frozen peripheral blood mononuclear cells (33). Special care should be taken to compare fresh with frozen samples, and as good practice it is strongly recommended to perform preliminary experiments to verify the quality/status of each antigen of interest before and after freezing.
4) *Systematic controls to monitor analyzer performance*. Flow cytometers are subject to laser wear and fluidic instability over time. To compare samples acquired in different days, several controls should be used to ensure the correct and constant performance of flow cytometer and the consistency of data collection. Indeed, some analyzers are equipped with a system that performs daily electronic checks and automatically adjusts internal parameters.

Furthermore, reference stabilized blood samples with defined ranges of the main lymphocyte subsets are available to be used as controls, helping to avoid batch effects.

Once the samples have been acquired and processed in the proper way, the next step is gating. There are several ways to gate samples:

 -Manual
 -Semi-automatic
 -Automatic

Each method has advantages and disadvantages; for instance, if, on the one hand manual gating is time-consuming and operator-dependent, on the other it allows the analysis of very rare cell populations that are difficult to identify using automatic strategies. Automatic methods (briefly described in the previous section) use algorithms to systematically identify cell populations, thus avoiding operator-dependent inaccuracies. They can be further divided into “hypothesis dependent”, if the scientist sets specific cell subtypes to be measured, and “agnostic”, which are not based on specific hypotheses, allowing the identification of previously unknown cell cluster which could be missed by using manual gating approaches.

Following gate positioning, each cell population (both newly identified and already known) can undergo three types of measurements:

 a) Relative count
 b) Absolute count
 c) Fluorescence intensity
a) The relative count corresponds to the ratio between cell types that could be hierarchically dependent (e.g., percentage with respect to parental and grand parental cell population, such as percentage of CD4 with respect to T cells) or independent (e.g., ratio between T and B cells).
b) The absolute or actual count corresponds to the number of cells per volume (generally expressed as cells/ul or cell/mm^3^). In human blood, the necessary condition to obtain absolute counts is to process fresh non-washed samples and use either analyzers able to calculate the absolute number of cells based on sample volume or a fixed number of counting beads to be added to each sample. In the latter case, it is necessary to apply a simple proportion between number of beads and cells acquired to obtain actual counts. Alternatively, it is also possible to obtained actual counts from frozen samples if the leukocyte (or lymphocyte) count measured on the day of the withdrawal is combined with the relative counts obtained by flow cytometry from frozen material.
c) Generally defined as mean or median fluorescence intensity (MFI), it represents the expression level of an antigen (such as CD4, CD8, CD40, CD28) on a cell type (**Figure 3C**). A necessary condition to properly analyze MFIs is that the marker measurement in the specific cells follows a normal (Gaussian) distribution. For instance, CD4 expression measured in total T cells (which include an important amount of CD4 negative cells) is inaccurate because a bimodal distribution would be observed: one peak corresponding to CD4 negative cells and a second peak corresponding to positive cells (**Figure 3D**). In this case, the bimodal distribution does not mirror the expression level of CD4 positive cells; rather, it correlates the number of cells present in the first (negative) peak with respect to the second (positive) one (**Figure 3D**). Thus, CD4 MFI should be assessed only in the CD4 positive cells, where its distribution is normal. In additional cases, such as CD8 expression in T cells, the presence of three peaks is frequently observed, corresponding to negative, intermediate (dim), and high (bright) antigen expression. The negative peak should be excluded, while the expression of CD8 in the two positive peaks should be measured separately, especially if the number of CD8 dim T cells is consistently represented (**Figure 3B**). Also, the number of events in which the MFI is measured is very important to obtain reliable data, as the MFI of a few events is not very robust. Thus, also in this case, the general rule is that the more events acquired, the more robust the MFI data are (**Figures 3E, F**).

## Understanding Causal Effects of Immune Cell Levels in Human Disease: The Hypothesis-Generating *vs* Hypothesis-Driven Approach

The comparison of specific immune cell levels between cases and controls has been a widely used approach to identify those cells or derived parameters that are more frequent in cases, and thus putatively predisposing to the disease, compared to controls. By contrast, those that are higher in controls are putatively protective for the disease. However, this case-control, hypothesis-driven comparison of immune phenotypes is limited by *a priori* knowledge and is also affected by second order effects due to the disease process and the administered therapy. That can lead to mistaken inference of a consequence of a disease for a cause (so-called *reverse causation*).

A more robust and systematic approach to identify immune cell traits implicated in the disease process relies on correlations between genetic association signals detected in different sample sets. This hypothesis-generating approach first establishes, *via* quantitative trait locus (QTL) GWAS, the genetic control of as many immune cell traits as possible in as many general population individuals as possible. The resulting association signals for immune cell traits are then evaluated for any significant overlap with association signals from GWAS on autoimmune disease risk, typically performed with a case-control design. The increasing availability of autoimmune disease GWAS summary statistics offers valuable resource data to search for such overlaps, which can then be formally demonstrated using specific statistical approaches like *co-localization* methods. These allow to formally test whether two association signals at the same locus for two different traits or diseases share the same causal variant (34). In principle, if a gene variant X is causally related to both a quantitative immunophenotype Y and an autoimmune disease Z, it is possible that the immunophenotype Y is involved in the process leading to the autoimmune disease Z and represents an endophenotype for that disease.

**Figure 5:**
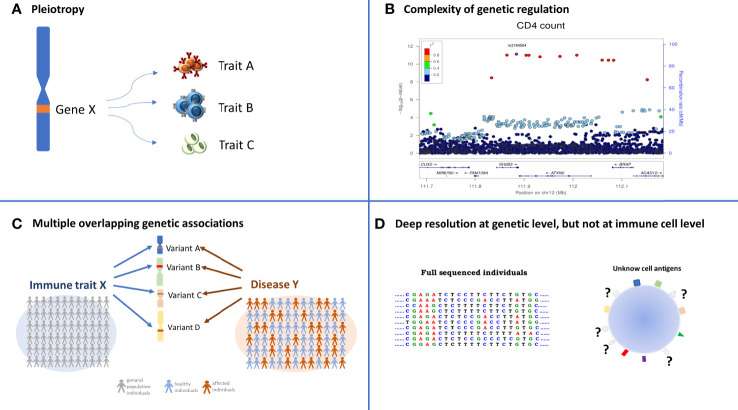
Complexity in identifying overlapping association between immune trait and diseases. The four quadrants summarize the layer of difficulties that must be considered when the overlapping association approach is applied. **(A)** Pleiotropy: a specific variant can regulate several traits; **(B)** the identification of the causal variant is often not immediate due to the high LD among variants. Each dot represents a genetic variant, LD among variants (expressed as r^2^) is color coded and specified in the legend, the significance of the association, expressed as −log10[P value], is indicated in the left y axis, and the genomic positions in the x axis; **(C)** the identification of several independent genetic variants associated with the same immune cell X and the same disease Y (multiple overlapping genetic association) requires thousands of deeply immune profiled individuals, both from general populations to dissect immune cell genetic regulation, and from case-control studies to identify genetic regulation of diseases; **(D)** the genomic information of each individual is very deep being at single-base resolution, whereas the knowledge of all the proteins expressed on cell surface is far from being complete.

The route toward unequivocally linking a given immune cell variable with one or more immune mediated disease is rather complex and hindered by several factors including pleiotropic effects, low statistical power and incomplete characterization of immune cell variation, as follows (**Figure 5**).

Pleiotropy (**Figure 5A**), a phenomenon in which one genetic locus influences two or more phenotypic traits (35), is an emerging feature of current GWAS results that can complicate the resolution of the causal-relationships to a true disease-related intermediate immune phenotype (3). It is classically divided into biological or mediated, with the former referring to a genetic variant that has a direct influence on the regulation of more than one trait and the latter occurring when a variant directly influences one trait, which in turn influences another trait. Pleiotropy can also be spurious, which is due to various design artifacts that cause a genetic variant to appear fallaciously associated with multiple traits.

During the last decade, 93 loci associated with immune cells traits have been identified by genome wide association studies (4, 5, 36–38), and about half of these loci overlap with previously reported disease-associations predominantly for autoimmune disorders. Most of the detected genetic signals were characterized by pleiotropy; 61% of these signals regulate protein levels on the cell membrane (MFIs), whereas only 25% and 14% of them were found associated with relative and absolute counts, respectively (3). This can likely occur either because of the common origin and shared mechanisms of genetic regulation of different immune cells or because of the interrelated functions of many immune cell types, with some cells controlling the level of other cells. And the complexity of genetic associations detected so far with the genetic regulation of immune traits goes beyond the detection of pleiotropic effects and includes several instances of multiple independent signals in a given gene region affecting the same cell or protein expression, and in other cases unrelated traits (**Figure 5B**, also see the CD25 example in the next section).

In the presence of strong pleiotropy, approaches that exploit Mendel’s second law of inheritance to search for *multiple independent genetic associations* associated with both the same intermediate immune phenotype and autoimmune disease outcome provide a route to somewhat restrict the number of coincident associations to those most likely involved in disease pathogenesis. Indeed, if two or more independent genetic signals are simultaneously associated with the same disease predisposition and a specific quantitative trait, with a coherent reduction or increase in the trait levels, it is more probable that the trait is causally implicated in the disease predisposition (**Figure 5C**) (1, 3). This approach can help identify the most promising association signals to follow up with downstream functional studies; however, it does not reveal the presence of confounding factors regulating both trait and disease, because it is based on a simple comparison among a few association statistics. For these reasons, *Mendelian randomization* (MR) is now the approach most frequently applied to infer a causal relationship between a quantitative trait (defined as *exposure*) and a disease (*outcome*). The genetic variants associated with a quantitative trait are used as instrumental variables (Ivs) to test the causal relationship between exposure and outcome. Critically, because they are constant, they are not affected by reverse causation and/or confounders. Like the methods previously described, this approach is essentially based on the summary statistics for a set of Ivs chosen to satisfy specific hypotheses, such as the association with exposures, to which appropriate statistical regression methods are applied (39–41). The increasing availability of large datasets and the consequent increasing number of variants that can be tested are facilitating the application of the MR approach.

Another limiting factor in making causal inferences about the involvement of a given immune cell in a particular autoimmune disease is the relatively small sample size of the immune cell GWAS performed to date, which constrains the generation of robust instrumental variables for Mendelian randomization approaches. Furthermore, the true disease-related cell type may not even have been assessed in immune cell trait GWAS! The latter limitations can be overcome thanks to the development of more advanced cytofluorimeters and the implementation of automation methods to permit considerable enlargement of the immune-phenotypic space (**Figure 5D**) examined in an increasingly larger number of individuals.

## Therapeutic Targets, Multi-Specificity, and Personalized Medicine

After establishing co-localized association signals between immune cell traits and autoimmune disease risk that are likely to share a causal variant pointed by Mendelian randomization approaches, a critical step toward the identification of the right therapeutic targets is to identify the DNA variant, and establish/infer the protein product, underpinning such overlapping associations that could be modulated therapeutically.

In short, an initial strategy commonly applied to statistically exclude all but ideally one or a few polymorphisms as causal variants in GWAS-associated regions encompasses several methods known collectively as “fine mapping” (42). This strategy requires an unbiased, and as comprehensive as possible, ascertainment of genetic variation -through large-scale DNA sequencing and the use of informative imputation panels- to split the genetic contributions of individual variants in an associated region, allowing prioritization of those with the highest probability of being causal. The most plausible causal polymorphisms present in the so-called “credible set” are then ranked using several metrics, including sequence conservation across species and functional genomic data (such as transcription factor binding), which produce a score predicting functional relevance. Unfortunately, even after these methods have been applied, the genetic resolution of association signals to a single-variant, single-gene may still be limited by several factors. These include the strong linkage disequilibrium (non-random association of alleles at different loci in a given population) (43) between several candidate variants that in extreme cases may be so closely related as to be genetically indistinguishable (because they always co-occur in the same individuals). An additional difficulty which hampers variant functional annotation, arises from the fact that the vast majority (~80%) of lead variants of association signals with immune traits are localized in “non-coding regions” of the genome with only a fraction of them altering known sequence motifs of transcription factors (3, 10, 44), thus not easy to interpret, even though they must play a very relevant role in gene expression regulation. Most importantly, even statistical refinement of the association signal to a single putative causative DNA variant does not in itself indicate that the gene harboring is causative. In fact, there are multiple examples of long-range control of gene expression by variants located in neighboring genes detected through technologies such as promoter capture with “Hi-C” (45).

Still, despite these difficulties, the identification of the causal genes highlighting their products as therapeutic targets can be often achieved through expression quantitative trait loci (eQTLs, based on the analysis of the influence of genetic variation on RNA levels) and/or protein QTLs (pQTLs, based on the analysis of the influence of genetic variation on protein levels), which in the cytofluorimetric studies are represented by the expression level of immune cell protein levels (MFIs). In addition to *cis* effects, these analyses can reveal *trans* effects, i.e., trans pQTL and eQTL associations that highlight protein targets for therapeutic intervention encoded by genes located far away, and even on different chromosomes, from the variant/gene underlying the primary association signal but whose expression is affected by it or its protein product or a nearby genetically related variant.

The utility of pQTL and eQTL analyses extends to the determination of the effective direction of the association. This is inferred from the direction of change in levels of gene products associated with disease risk – for example, evaluating whether a disease-protective allele (whose effect we want to therapeutically reproduce) decreases or increases transcript levels of a gene or corresponding protein. This is thus a critical step because it informs the direction (inhibition/stimulation) of therapeutic modulation of the target.

Such analyses are facilitated by the rapidly growing number of large datasets annotating information that can systematically help to bridge GWAS associations to expression levels. One key resource is the Genotype-Tissue Expression (GTEx) catalogue, providing eQTL analysis for 49 human tissues in 838 individuals (46). Additional sources to help assess the impact of regulatory variants include databases, such as the Human Induced Pluripotent Stem Cell Initiative (HipSci) (47) reporting mutations in reprogrammed induced pluripotent stem cell and LINkage Disequilibrium-based Annotation, LinDA brower (http://linda.irgb.cnr.it) that provides annotations and statistics for the query variant and for variants in linkage disequilibrium with the query. While these comprehensive public resources to study tissue-specific transcription and expression are essential to identify target genes and direction of effects of associations signals with immune cells and other trait types, GWAS results may in turn give rise to more targeted studies of transcription and regulation to elucidate the fine mechanisms of gene expression at specific loci. As an example, in a GWAS analysis it was uncovered the association of multiple sclerosis and systemic lupus erythematosus with a genetic variant in the 3’UTR of the *TNFSF13B* gene, which encodes the cytokine B-cell-activating-factor (BAFF) (10). The same signal also correlated with increased circulating B cell and immunoglobulin levels, giving a potential mechanistic explanation for the disease association. The causal variant underlying these associations was found to be an insertion-deletion (GCTGT > A, [GCTG/-] where the minor risk-associated allele A (referred as ‘BAFF-var’) was predicted to create an upstream alternative polyadenylation site (APA). This APA was experimentally demonstrated and the resulting shorter transcript, BAFF-var mRNA, was more actively translated than the long wild-type mRNA (BAFF-WT) partly because it lacked a site of repression by microRNA miR-15a (10). Subsequent analyses showed that the short 3’UTR lacked also a binding site of repression by the RNA Binding Protein (RBP) NF90 and revealed that, in the BAFF-WT mRNA, NF90 suppresses BAFF production by promoting the interaction of miR-15a with BAFF-WT mRNA. As a consequence of this lack of repression of BAFF expression due to BAFF-var, soluble BAFF is produced at higher levels determining a cascade of immune events leading to increased risk for systemic lupus erythematosus and multiple sclerosis (48). It is expected that this type of fine analysis of the regulation of gene expression will increasingly contribute to a detailed understanding of the molecular mechanisms of genetic associations with immune traits.

The obvious next critical step toward the therapeutic modulation of a protein target identified with genetic approaches is the assessment of its druggability – that is, its susceptibility to be potentially modulated in its effects by drug-like small molecules (typically targeting hydrophobic pockets) or by so called “biologicals” (more commonly targeting extracellular domains such as those of receptor proteins or soluble molecules) or by new molecular approaches, such as those based on small interfering RNA, antisense oligonucleotides, mRNA delivery, gene editing with CRISPR–Cas9, and PROteolysis-TArgeting Chimaeras (PROTAC) (49–51).

In particular, the protein target identification approach presented here, built on the results of flow cytometry coupled with genetic data, offers an obvious opportunity for therapeutic intervention through the generation of biological products, specifically, as we detailed below, through a new class of poly-specific antibodies. In contrast, many current monospecific antibody-based therapies aimed to block, or in few cases enhance, the activity of a single antigen generally expressed on the cell surface membrane, such as anti-CD28, CD40, and CD25. Nevertheless, these mono-specific drugs are affected by poor cell specificity causing reduced efficacy and predisposition to side effects like increased risk of other autoimmune diseases. Indeed, targeting broadly expressed markers such as CD25 or CD27, which are expressed in both T and B cells, or CD28, expressed in both CD4 and CD8 T cells, could cause unspecific blocking of this marker in cells that are not involved in a specific disease (3).

**Figure 6:**
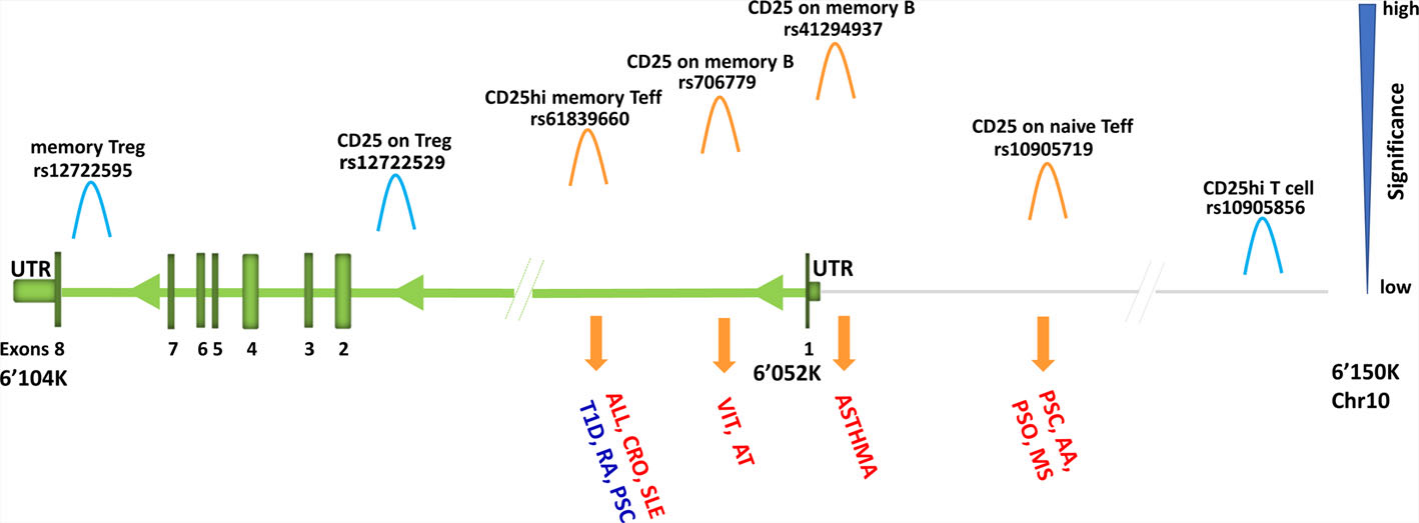
Association signals at *IL2RA* region. Representation of *IL2RA* gene (green) and about 100 kb upstream to the gene (grey line). The association signals with immune cell traits are depicted by «hills» which are colored in orange or light blue if overlapping or not overlapping with disease-association signals, respectively. Disease is in red if the predisposing allele is associated with increase of immune cell traits, whereas it is in blue if the predisposing allele is associated with decrease of immune cell traits. Disease acronyms: T1D, type one diabetes; RA, rheumatoid arthritis; PSC, primary sclerosis cholangitis; ALL, allergy; CRO, Crohn’s disease; SLE, systemic lupus erythematosus; VIT, vitiligo; AT, autoimmune thyroiditis; AA, alopecia areata; PSO, psoriasis; MS, multiple sclerosis.

For instance, IL2RA, also known as CD25, encodes the alpha chain of IL-2 receptor and is expressed in regulatory T cells (Tregs), activated effector T cells, but also in B cells. In 2013, measuring CD25 in T cells only and using about 8.2 million variants, an overlapping association between CD25hi effector T cells and type 1 diabetes was found in the *IL2RA* region (1). More recently, by increasing both the cell types where CD25 has been assessed and the number of interrogated variants, seven independent signals in the *IL2RA* locus (all regulating CD25 expression) were identified (3) (**Figure 6**). Some signals were T cell specific; others were B cell specific, still, others involved both T and B cells. Four out of the seven independent signals detected in this region were associated with immune-diseases and pointed to different traits, in some cases with opposite direction of effect, potentially leading to adverse therapeutic complications (**Table 1**). In more details, the inhibition of T cells expressing high levels of CD25 may be efficacious in Crohn’s disease, but harmful in type 1 diabetes and juvenile rheumatoid arthritis for which a stimulation of the same cells is likely to be effective. These data also suggest that reduction of CD25 on naive effector helper T cells could be an effective therapy in multiple sclerosis and alopecia areata. But inhibition of CD25 on a specific subset of memory B cells called late memory B cells (identified as positive for CD19, but negative for IgD and CD38) could be useful in vitiligo and autoimmune thyroiditis therapies (**Figure 6**).

**Table 1:** Immune traits associated with diseases *via* overlapping genetic association and having opposite direction of effect in different diseases. Extracted from Orrù et al., 2020 (3).

Cell trait name	Primary drug targets	Disease	Proposed therapeutic modulation of primary drug targets	Expected increased risk for other autoimmune disease (side effect)
CD32 on monocyte	CD32	CRO, IBD, KD, AS, UC	inhibition	SLE
CD32 on monocyte	CD32	SLE	activation	CRO, IBD, KD, AS, UC
CD28 on CD39+ CD4+	CD28	UC, CEL	activation	MS
CD28 on CD39+ CD4+	CD28	MS	inhibition	UC, CEL
CD28 on CD4+	CD28	UC, CEL	activation	MS
CD28 on CD4+	CD28	MS	inhibition	CEL, UC
CCR2 on monocyte	CCR2	BD, CEL	activation	SLE
CCR2 on monocyte	CCR2	SLE	inhibition	CEL, BD
HLA DR on CD14- CD16+ monocyte	HLA DR	CEL, Allergy, MS, Cutaneous squamous cell carcinoma	inhibition	VIT, AA
HLA DR on CD14- CD16+ monocyte	HLA DR	VIT, AA	activation	CEL, Allergy, MS, Cutaneous squamous cell carcinoma
CD80 on myeloid DC (especially CD62L+)	CD80	CEL	inhibition	CRO, IBD, Allergy
CD80 on myeloid DC (especially CD62L+)	CD80	CRO, IBD, Allergy	activation	CEL
CD45RA on naive CD4+	CD45RA	Allergy, MS	inhibition	RA
CD45RA on naive CD4+	CD45RA	RA	activation	MS, Allergy
CD25hi%CD4+ (especially CD25hi CD45RA- CD4 not Treg %CD4+)	CD25, CD4, CD3	T1D, PSC, JIA	activation	Allergy, CRO
CD25hi%CD4+ (especially CD25hi CD45RA- CD4 not Treg %CD4+)	CD25, CD4, CD3	Allergy, CRO	inhibition	T1D, PSC, JIA
CD25 on CD45RA- CD4 not Treg	CD25	T1D, PSC, JIA	activation	Allergy, CRO
CD25 on CD45RA- CD4 not Treg	CD25	Allergy, CRO	inhibition	T1D, PSC, JIA
CD25 on CD4+	CD25	T1D, PSC, JIA	activation	Allergy, CRO
CD25 on CD4+	CD25	Allergy,CRO	inhibition	T1D, PSC, JIA
CD25++ CD8br%Tcells	CD25, CD8	T1D, PSC, JIA	activation	Allergy, CRO
CD25++ CD8br%Tcells	CD25, CD8	Allergy, CRO	inhibition	T1D, PSC, JIA
CD11c on myeloid DC	CD11c	IgAN	activation	SLE
CD11c on myeloid DC	CD11c	SLE	inhibition	IgAN
CD19 on B cell (especially sw mem IgD-CD27+)	CD19	IBD, CRO, Ob	activation	T1D
CD19 on B cell (especially sw mem IgD-CD27+)	CD19	T1D	inhibition	CRO, IBD, Ob
IgD+ AC	IgD, CD19/CD20	Allergy, Asthma, ALL, AM	inhibition	CC, CRO, PBC, RA, T1D, UC, Bronchial hyperresponsiveness in asthma, Selective IgA deficiency, Liver biliary cirrhosis
IgD+ AC	IgD, CD19/CD20	CC, CRO, PBC, RA, T1D, UC, Bronchial hyperresponsiveness in asthma, Selective IgA deficiency, Liver biliary cirrhosis	activation	Allergy, Asthma, ALL, AM
Unsw Mem (IgD+CD27+) %lymphocyte	IgD, CD27, CD19/CD20	HBVI, CRO, IBD, MS, SLE	inhibition	RA, Depression, KD
Unsw Mem (IgD+CD27+) %lymphocyte	IgD, CD27, CD19/CD20	RA, Depression, KD	activation	HBVI, CRO, IBD, MS, SLE
CD27 on memory B cell (especially IgD-CD38dim)	CD27	HBVI, CRO, IBD, MS, SLE	inhibition	RA, KD
CD27 on memory B cell (especially IgD-CD38dim)	CD27	RA, KD	activation	HBVI, CRO, IBD, MS, SLE
CD40 on B cell (especially IgD-CD27-)	CD40	HBVI, CRO, IBD, MS, SLE	activation	RA, KD
CD40 on B cell (especially IgD-CD27-)	CD40	RA, KD	inhibition	HBVI, CRO, IBD, MS, SLE
IgD- CD27- %B cell	CD19/CD20	HBVI, CRO, IBD, MS, SLE	activation	RA, KD
IgD- CD27- %B cell	CD19/CD20	RA, KD	inhibition	HBVI, CRO, IBD, MS, SLE

Overall, the genetic associations observed in the *IL2RA* region can predict the efficacy and potential adverse effect of the broad blocking of CD25 that causes a reduction of CD25 activity in cells not implicated in disease predisposition (e.g., Tregs).

A similar scenario was observed for several antigens, such as CD32, CD28, and CD40, whose increase is associated with predisposition to some diseases, but also with protection from others (**Table 1**). But if the targeted antigen can be addressed in specific cells (for instance either B or T cells in the case of CD25), the adverse effects should be minimized. Thus, the generation of multi-specific drugs, able to recognize more than one antigen simultaneously, can provide an optimal way to ensure specificity and reduce adverse effects.

Multi-specific drugs are in clinical trials especially for cancer treatment, where an antibody binds immune cells such as CD3-positive, while another antibody binds cancer cells, thereby redirecting T-cell cytotoxicity to malignant cells (52, 53). However, the same approach can be useful to engage two molecules on the membrane of one cell (*in-cis* binding). For instance, MGD010 is a dual-affinity retargeting (DART) protein which simultaneously binds the B cell surface proteins CD32B and CD79B to deliver a co-inhibitory signal that dampens B cell activation (54). The intended mechanism of MGD010 is to modulate the function of human B cells while avoiding their depletion and could be useful for treatment of rheumatoid arthritis and other autoimmune and inflammatory diseases. Notwithstanding, only few bispecific antibodies have been approved and marketed, namely blinatumomab (55), simultaneously targeting B cell CD19 antigen and T cell CD3 antigen against B cell malignancies, and emicizumab (56, 57), targeting coagulation factors IXa and X against hemophilia A. Finally, catumaxomab, approved in Europe for the intraperitoneal treatment of malignant ascites, binds to the epithelial cell adhesion molecule (EpCAM), T cells (*via* CD3), and to accessory cells, including dendritic cells, macrophages, and natural killer cells through its Fc-fragment (58). Approved in 2009, catumaxomab was however withdrawn from the US market in 2013 and from European market in 2017 when the company became insolvent.

More recently, tri-specific drugs have been developed. Among them, a single molecule designed by Xu and colleagues is able to bind three HIV-1 envelope determinants: the CD4 binding site, the membrane proximal external region, and the V1V2 glycan site, showing higher potency and breadth compared to previously used antibodies and complete immunity against a mixture of simian-human immunodeficiency viruses (SHIVs) in nonhuman primates (59).

The reason why few multi-specific drugs have been created and approved is that they are not easy to generate due to their instability, low solubility, unwanted inter-subunit associations, and enhanced immunogenicity (60). The evaluation of these therapeutic properties as well as manufacturability and safety profile is called developability.

Another relevant consideration is the choice of the most appropriate dose. Several studies demonstrated that even when a drug is able to ameliorate a disease condition, its administration at a wrong concentration can cause potentially deadly side-effects. This happened in 2006 when six healthy young males were enrolled in the first phase 1 clinical trial of the CD28 super-agonist TGN1412, which can activate T cells, particularly regulatory T cells, thus potentially efficacious against autoimmunity where a reduced function of Tregs is expected. All volunteers had an unpredicted multiple cytokines release syndrome and underwent intensive cardiopulmonary support, dialysis, and administration of both a high-dose of anti-inflammatory drugs such as methylprednisolone and an anti–interleukin-2 receptor antagonist antibody. Fortunately, all six volunteers survived (61). It was clear that the drug activated effector T cells instead of Tregs. Some years later, the reasons for the preclinical study failure of TGN1412 were found (62). Firstly, only about 2% of T cells circulate in the peripheral blood (63), thus human T cells used for *in vitro* studies (which derive from that 2%) respond differently compared to those *in vivo*, which include also the remaining 98% of T cells. Secondly, in mouse models living in a germ-free animal house used to test the drug, CD4 effector memory cells are much lower in numbers and easily controllable by TGN1412-activated Tregs compared to humans. Thirdly, in cynomolgus macaques, also used to test the drug, CD4 effector memory cells down-regulate CD28, and thus it cannot bind TGN1412; this does not occur in humans. In 2014, Tabares and colleagues (64) demonstrated that a strong reduction of TGN1412, now renamed TAB08, accompanied by the administration of corticosteroid drug (such as methylprednisolone), activates Tregs without a cytokine storm, thus making it useful in rheumatoid arthritis and other autoimmune therapies.

The TGN1412 results exemplify the need to identify the correct dose for the correct target. Notably, the proper dose could also depend on our genome, indeed, differences in our DNA sequence that affect the levels of the drug target (such as specific cell type or protein) could modify the efficacy of the pharmacological treatments, thus an individual could need a different drug concentration compared to another individual - a type of personalized medicine.

## Concluding Remarks

Flow cytometry combined with systematic GWAS of immune traits in general population cohorts and case-control GWAS data on autoimmune disease risk is a powerful strategy to identify specific proteins, cells, and pathways involved in the etiopathogenesis of immune related diseases. After appropriate validation with functional studies, this strategy will be increasingly relevant to identify therapeutic targets and reinforce causal relationships as the technology will evolve to permit a considerable expansion of the number of markers assessed simultaneously by flow cytometry and of the sample size of the studies. Corresponding advances in the generation of a new class of *in-cis*, multi-specific antibodies to engage these targets will progressively increase efficacy and minimize the potential side effects in the treatment of autoimmune diseases.

In summary, from flow cytometry data collection to drug therapy development, four main steps are relevant:

- coupling flow cytometry data to genetics in the general population sample set to identify the genetic component driving the interindividual immune variability;
- systematically searching for overlapping association between immune trait-associated variants (from population-based datasets) and disease-associate variants (from case-control datasets);
- causality confirmation of identified disease risk variants through functional studies;
- drug development in a cell-specific context.

## Author Contributions 

All authors contributed to the article and approved the submitted version.

## Funding

The preparation of this manuscript was supported by the Intramural Research Program of the National Institute on Aging, National Institutes of Health (NIH) (contract HHSN271201600005C), the Horizon 2020 Research and Innovation Program of the European Union (grant 633964), and Fondazione di Sardegna (grant U1301.2015/AI.1157. BE Prat. 2015-1651).

## Conflict of Interest

The authors declare that the research was conducted in the absence of any commercial or financial relationships that could be construed as a potential conflict of interest.

## Publisher’s Note

All claims expressed in this article are solely those of the authors and do not necessarily represent those of their affiliated organizations, or those of the publisher, the editors and the reviewers. Any product that may be evaluated in this article, or claim that may be made by its manufacturer, is not guaranteed or endorsed by the publisher.
